# Life Cycle Simplifications in Aphids Drive Changes in Evolutionary Rates and Selection Regimes

**DOI:** 10.1093/molbev/msaf307

**Published:** 2025-12-01

**Authors:** Théo Vericel, Gaorui Gong, Fabrice Legeai, Aurélie Etier, Julie Jaquiéry, Jean-Christophe Simon

**Affiliations:** UMR 1349 IGEPP, INRAE, Le Rheu 35650, France; UMR 1349 IGEPP, INRAE, Le Rheu 35650, France; UMR 1349 IGEPP, INRAE, Le Rheu 35650, France; UMR 1349 IGEPP, INRAE, Le Rheu 35650, France; UMR 1349 IGEPP, INRAE, Le Rheu 35650, France; UMR 1349 IGEPP, INRAE, Le Rheu 35650, France

**Keywords:** life cycle transition, comparative genomics, host alternation, aphids, dN/dS

## Abstract

Transitions toward simplified life cycles can reshape evolutionary trajectories, yet their impact on the rate of molecular evolution remains poorly understood. In aphids, host alternation (heteroecy) entails obligate seasonal migration between highly distinct plant hosts—typically woody and herbaceous species—and has been repeatedly lost, giving rise to monoecious species with simplified life cycles. Using comparative genomics across 46 aphid species, we tested whether transitions from heteroecy to monoecy alter evolutionary dynamics at the gene level. We identified 9,304 orthologs and estimated evolutionary rates (dN/dS) and shifts in selection regimes in the diverse Aphidinae subfamily. We found that 715 orthologs evolved faster in monoecious species, primarily due to relaxed selection, while heteroecious species showed signatures of intensified selection. Genes under relaxed selection in monoecious species were enriched for functions related to environmental sensing, signaling, nutritional adjustments, morph determination, and migration related—traits likely central for host alternation. These results suggest that the loss of a complex life cycle leads to reduced selective constraints as a consequence of ecological simplification. This study provides a robust evolutionary framework for understanding how life cycle transitions shape molecular evolution and drive gene decay following trait loss.

## Introduction

Life cycle transitions occurring within a single organism's lifetime, such as shifts between environments (eg aquatic to terrestrial habitats, alternation between different hosts; Dixon 1998; Bonett and Blair 2017) or between developmental stages (larva to adult; McEdward 2000), impose constraints on the genome. Individuals must possess a genetic toolkit capable of supporting distinct functional requirements across multiple life stages or environmental conditions (Moran 1994).

Evolutionary transitions toward simplified life cycles, such as the loss of the typical adult phase in paedomorphic species (Liedtke et al. 2022) can therefore profoundly affect genomic architecture. Traits that were once essential may become obsolete and are no longer expressed. Similarly, shifts to novel environments, as for example the transition to an aquatic lifestyle in mammals (Farina et al. 2024), the evolution of parasitism in ants (Schrader et al. 2021), or of endosymbiosis in bacteria (Siozios et al. 2024), can render previously vital genes useless in the new ecological context. In both scenarios, these genes are freed from selective constraint, allowing them to decay or acquire new functions. Importantly, both life cycle simplification and environmental shifts are frequently accompanied by changes in effective population size (eg Tong et al. 2022), which in turn have genome-wide consequences, influencing the tempo and trajectory of evolution.

Despite their importance, our understanding of how such transitions affect molecular evolution, genetic innovation, and diversification remains limited (Liedtke et al. 2022; Eastment et al. 2024; Peoples et al. 2025; Zhao et al. 2025). This knowledge gap restricts our ability to fully grasp the mechanisms driving macroevolutionary patterns and the adaptive potential of species in response to environmental change.

Understanding life cycle transitions is particularly relevant in organisms that exhibit complex alternations in behavior, morphology, and ecological niches. One striking example is host alternation—or heteroecy—in aphids, a compelling system for exploring how such transitions can drive or constrain evolutionary changes. In this complex life cycle, aphids must complete seasonal migrations between highly divergent host plants (Dixon 1998; Emden and Harrington 2007). These obligatory migrations involve the production of specialized winged morphs in response to environmental cues, along with physiological and behavioral changes that enable individuals to locate, recognize and successfully feed on distinct plant types. Typically, heteroecious aphid species alternate between a woody primary (winter) host, where sexual reproduction and oviposition occur, and an herbaceous secondary host, where asexual reproduction predominates from summer to early fall (Dixon 1998; Emden and Harrington 2007).

Although only about 10% of the ∼5,000 described aphid species are heteroecious, host alternation is phylogenetically widespread—it is found in every family of the aphid superfamily (Aphidoidea) and across numerous subfamilies (von Dohlen and Moran 2000). Remarkably, both monoecious and heteroecious species often coexist even within the same genus (Dixon 1998), indicating a high degree of evolutionary lability. This widespread but uneven distribution suggests a complex evolutionary history involving numerous transitions between heteroecy and monoecy throughout Aphidoidea evolution. Yet, despite its ecological and evolutionary significance, the history of heteroecy in aphids remains unresolved, hampered by gaps in phylogenetic resolution (Jousselin et al. 2024) and limited understanding of the genetic and molecular mechanisms governing life cycle variation (Shih et al. 2023). There is still no consensus on how many times heteroecy has independently evolved in Aphidoidea, although a few independent origins are suspected (von Dohlen and Moran 2000; Ortiz-Rivas and Martínez-Torres 2010; Peccoud et al. 2010). Numerous studies have proposed that heteroecy may represent the ancestral state in the Aphidinae subfamily, which comprises 57% of all described aphid species, though conclusive evidence is still lacking (Blackman and Eastop 1984; Moran 1988, 1992; von Dohlen and Moran 2000; von Dohlen et al. 2006; Ortiz-Rivas and Martínez-Torres 2010). Many genera within Aphidinae exhibit a shared association with Rosaceae as a primary host, a pattern consistent, though not definitive, for an ancestral life cycle involving alternation between woody rosaceous and herbaceous plants (von Dohlen et al. 2006; Peccoud et al. 2010). From this putative ancestral condition, aphids appear to have diversified through a range of evolutionary trajectories, including host shifts and adaptation to novel plant lineages, loss of alternation, and potentially reacquisition of heteroecy. Genera such as *Brachycaudus, Cryptomyzus, Dysaphis,* and *Myzus* illustrate various stages along these pathways (Jousselin et al. 2010; Peccoud et al. 2010). The occurrence of a specialized morph—the gynopara, which returns to the primary host to produce sexual females—across all heteroecious Aphidinae suggests a shared evolutionary origin of host alternation (Moran 1988), although convergent evolution cannot be ruled out (von Dohlen et al. 2006). A Bayesian ancestral state reconstruction further supports a shared origin, estimating a 98.7% probability that heteroecy was ancestral in Aphidinae (Choi et al. 2018). Nevertheless, the ancestral status of host alternation remains inconclusive, with alternative evolutionary scenarios remaining plausible. More robustly supported is the pattern that losses of heteroecy have occurred far more frequently than gains within the subfamily (Hardy et al. 2015), implying that most extant Aphidinae likely descended from heteroecious ancestors (Moran 1990).

Another unresolved question concerns the evolutionary significance of host alternation in aphids, for which two main contrasting hypotheses have been proposed. The ecological optimization hypothesis posits that heteroecy evolved due to adaptive advantages conferred by alternating between hosts, such as improved access to seasonal resources or refuges (Mackenzie and Dixon 1990, 1991). In contrast, the historical constraint hypothesis interprets heteroecy as a maladaptive trait, maintained by the inability to escape the primary host due to the specialization of first generation of aphids (Moran 1988, 1990). More recent phylogenetic analyses have tested these competing hypotheses, but have not yielded a definitive resolution. Instead, patterns suggest that the evolution of life cycle complexity in aphids may be lineage-specific, shaped by a combination of adaptive and historical factors (Hardy et al. 2015).

If heteroecy is ancestral, then monoecy likely represents a derived condition, involving the loss or functional decay of traits and genes required for host alternation. This would imply relaxed selection on such genes in monoecious species, potentially leading to their degradation via mutation accumulation. We therefore expect genes related to host alternation—particularly those regulating environmental cues sensing, morph differentiation, migration, and adjustments to unrelated host plants—to evolve at different rates in monoecious versus heteroecious species. If heteroecy is more evolutionarily constrained than monoecy, we would specifically expect stronger signatures of purifying selection on host alternation-related genes in heteroecious species. In this study, we tested whether evolutionary rates—more specifically the protein evolution rate measured as the ratio of nonsynonymous to synonymous substitutions (dN/dS)—differ between monoecious and heteroecious Aphidinae species, as genes involved in host alternation become nonessential in monoecious taxa. This approach not only offers insight into the evolutionary consequences of life cycle transitions at the molecular level but also provides an avenue to identify genes and functions underpinning host alternation. To achieve this, we assembled the most comprehensive dataset of complete aphid genomes and constructed a robust phylogenetic framework. We then estimated dN/dS ratios for orthologous genes across 30 Aphidinae genomes. Our analyses revealed genome-wide trends, most notably a relaxation of selective pressure in monoecious species, and highlighted candidate genes potentially controlling heteroecy in aphids.

## Results

### Distribution of Heteroecious and Monoecious Species on the Aphid Phylogenetic Tree

**Fig. 1.**
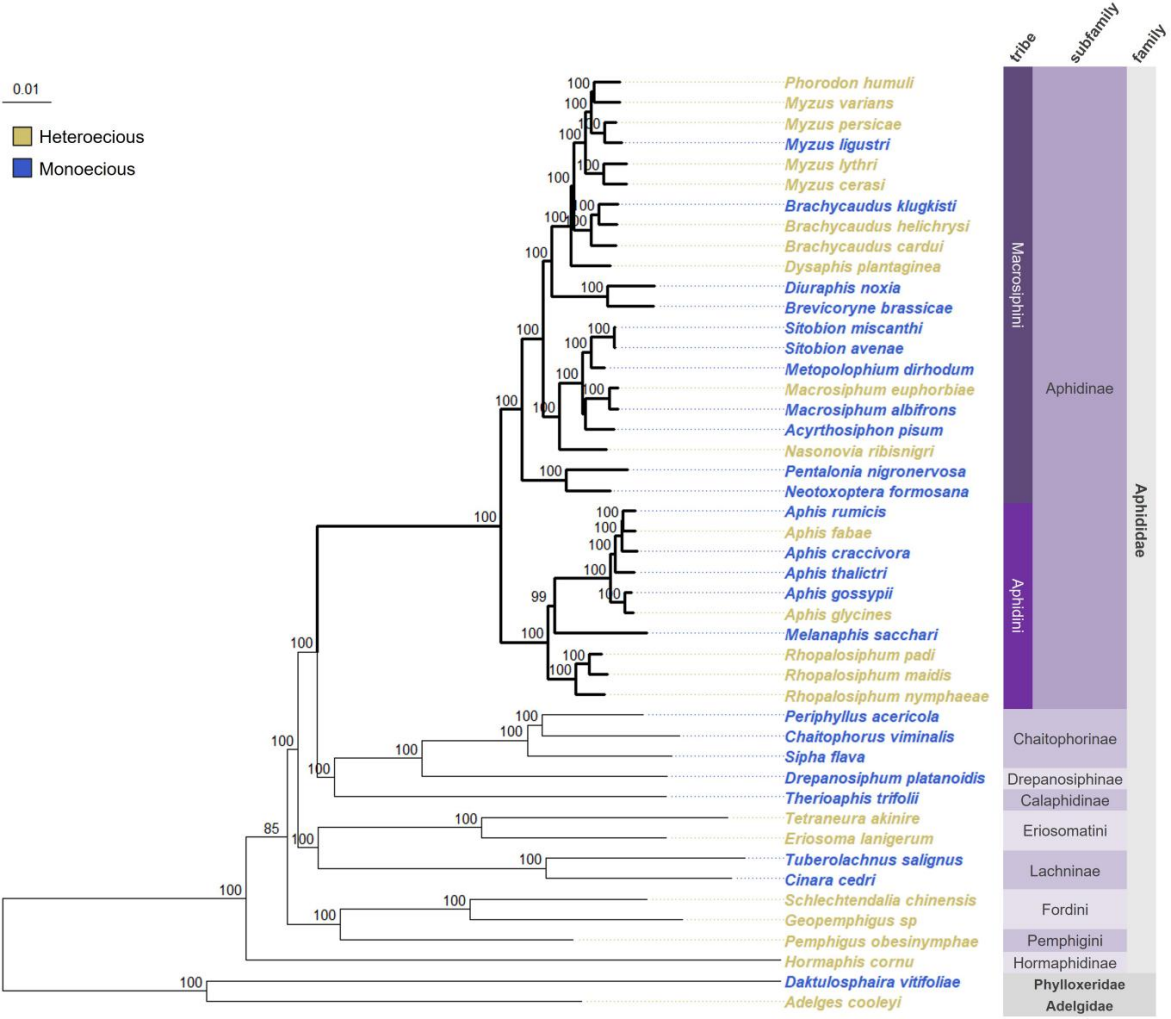
Maximum-likelihood phylogenomic tree (IQ-TREE) of aphid families inferred from a concatenation of 1491 orthologs present in the genomes of the 46 species. Bootstrap supports were obtained by 1000 replicates. Branches of the Aphidinae group used for dN/dS ratio analyses and selective regime characterization are shown in bold.

We reconstructed a comprehensive aphid phylogeny, by concatenating 1491 shared single copy orthologs derived from 46 available genomes (Fig. 1; Table S1). The phylogeny was supported by high bootstrap values, except for a single branch at deeper nodes, which connects the Hormaphidinae with the rest of the Aphididae subfamilies.

The phylogeny incorporated both heteroecious and monoecious species, which did not form distinct clades but instead coexisted within the same subfamily, tribe, and even genus. Focusing on the Aphidinae subfamily, which is the most extensively represented in our dataset, the phylogeny provided clear evidence of multiple transitions between heteroecy and monoecy.

### Identification of Orthologs With Different Evolutionary Patterns

**Fig. 2.**
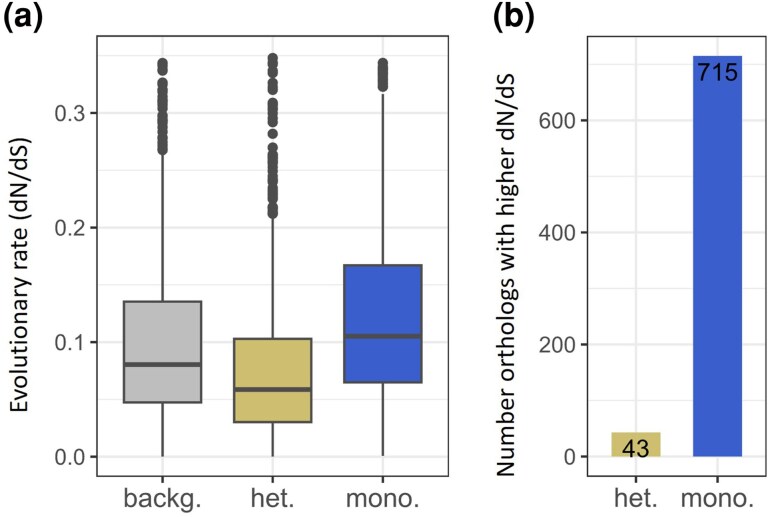
Estimated dN/dS ratios for the 758 orthologs that better fit the codeml M_1_ model, a model allowing different rates of evolution between terminal branches leading to monoecious and heteroecious species and background. a) dN/dS ratio estimated from M_1_ for the different parts of the tree (background [backg.], terminal branches leading to monoecious species [mono.] or heteroecious species [het.] for the 758 orthologs. b) Number of orthologs (among the 758) with dN/dS higher in terminal branches leading to heteroecious (or monoecious) species.

To test whether convergent patterns of evolution could be observed in certain orthologs depending on the life cycle type, we used Codeml from PAML 4.10.6 (Yang 2007) to compare dN/dS between heteroecious and monoecious species across a set of 9,304 single-copy orthologs inferred from 30 Aphidinae genomes. We hypothesized that orthologs involved in heteroecy—no longer essential in monoecious species—would exhibit higher dN/dS ratios in monoecious species due to relaxed selection. To test this, we compared Codeml models M_0_ (which assumes an identical dN/dS across heteroecious and monoecious terminal branches) and M_1_ (which allows different dN/dS ratios). This comparison revealed that 758 out of 9,304 orthologs fit the M_1_ model better (Table S2). The overall distribution of the dN/dS estimates for these 758 orthologs showed a general pattern of higher values for monoecious terminal branches (median = 0.1116), intermediate values for the background (median = 0.0849), and lower values for heteroecious terminal branches (median = 0.0605, Fig. 2a). Differences in dN/dS between the two life cycles resulted from a combination of an average increase in dN in monoecious terminal branches, but also a decrease in dS in this same group (Figure S1). Interestingly, the majority (94.33%) of the 758 orthologs showed an increase in dN/dS in monoecious species (Fig. 2b), highlighting a general trend toward a higher dN/dS in monoecious than in heteroecious species.

### Characterization of the Selection Regimes of Orthologs

**Fig. 3.**
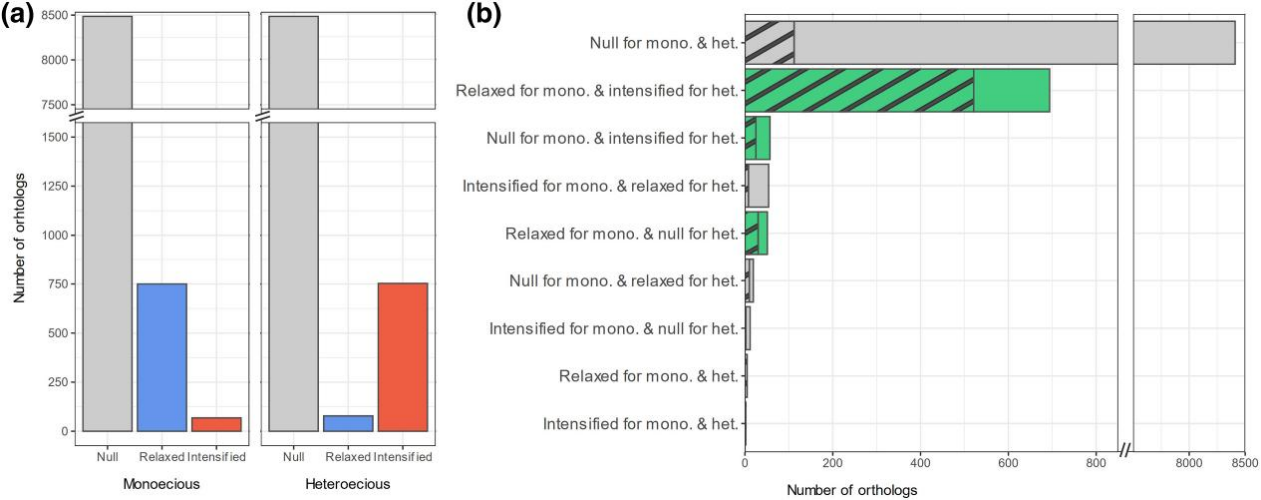
a) Number of genes that fit best the null model, or that show signs of relaxed or intensified selection when the RELAX test is carried out either on the monoecious (left panel, model M_M_) or heteroecious terminal branches (right panel, model M_H_). b) Intersection of the RELAX analyses on monoecious and heteroecious terminal branches, showing the number of genes (out of 9,304 orthologs) classified under different selection regimes for each life cycle type. Categories highlighted in green support the hypothesis of relaxed selection in monoecious species. Striped portions indicate the number of genes that fit the Codeml M_1_ model, with elevated dN/dS ratios in monoecious species. In both panels, the axis representing the number of orthologs is truncated respectively at 1500 (a) and 850 (b) to facilitate visualization.

The previous dN/dS analyses identified orthologs with elevated ratios in specific life cycle types but could not determine whether these increases resulted from relaxed or positive selection. RELAX 4.5 from HyPhy 2.5.63 (Wertheim et al. 2015), a branch-site model, was thus used to discriminate among these two scenarios. Note that RELAX infers selection in a relative manner; consequently, genes classified as “relaxed” in one branch set will typically appear “intensified” in the contrasting set. Thus, these analyses reveal differences between the tested branch sets rather than a temporal trajectory of selection intensity. RELAX allowed to identify 818 out of the 9304 orthologs fitting the M_M_ model better than the M_0_ model, evidencing a change in the selective regime on terminal branches leading to monoecious species compared to terminal branches leading to heteroecious species (Table S2). Interestingly, most of them (92%) were characterized as evolving under relaxed selection in monoecious species, and only a few under intensified selection (8%, Fig. 3a;  Figure S2). In contrast, the comparison of the M_H_ and M_0_ models revealed that of the 831 orthologs evolving differently in terminal branches leading to heteroecious species than from the rest of the tree, only 9% showed signatures of relaxed selection (Fig. 3a;  Figure S2). Most of them (91%) were identified as evolving under intensified selection. Excluding the 8,104 orthologs best explained by the M_0_ models for both life cycles, 89.7% of the remaining orthologs fall into three categories: (i) orthologs showing both relaxed selection in monoecious and intensified selection in heteroecious species (694 orthologs), (ii) orthologs showing relaxed selection in monoecious species only (51 orthologs), and (iii) orthologs showing intensified selection in heteroecious species only (57 orthologs) (Fig. 3b). These categories reflect selection patterns that support the hypothesis of reduced selection pressure in monoecious compared to heteroecious species. They encompass 576 out of the 715 orthologs showing higher dN/dS in monoecious species (Fig. 3b), reflecting the congruence between the RELAX and Codeml analyses, despite distinct methodological frameworks. Overall, these results revealed that heteroecy and monoecy are associated with opposite trends in terms of selection regime.

### Exploration of Ontology and Function of Candidate Orthologs

We then investigated the functions of the two sets of orthologs exhibiting shifts in selection regimes in monoecious aphid species, identified above, to uncover genes potentially involved in host alternation.

Among the 715 orthologs showing elevated dN/dS ratios in monoecious species, we found significant enrichment for four cellular component Gene Ontology (GO) terms: apical plasma membrane, basal plasma membrane, apical part of cell, and basal part of cell (Table S3). These terms were supported by 36, 20, 39, and 21 orthologs, respectively. There was considerable redundancy among these terms, with 45 orthologs associated with at least two of the terms and 11 linked to all four. One molecular function term, hydrolase activity acting on glycosyl bonds, was also enriched and supported by 19 orthologs (Table S3). After removing redundant and uncharacterized genes, a total of 61 annotated genes contributed to these enrichments. Functional annotation revealed that several genes are associated with environmental sensing/immunity (eg *Clock*-like, *mys*, *artichoke*, *Toll*-like receptors, Leulier and Lemaitre 2008; Ponton et al. 2023), neuronal function and signalization (eg *stan*, *uex*, *Lar*, *Fak*, *Epac*, *presenilin*, voltage-gated Ca²⁺ channel), and development (eg *Shroom*, *Diaphanous*, *Rho/Rap GTPases*, *vitellogenin-like lipoprotein*). Several genes potentially associated with aphid–plant interactions were also identified, including those implicated in digestion (eg *maltases*, *lysosomal alpha-mannosidase-like*), detoxification (eg *beta-glucuronidase*), and the transport of plant-derived compounds (eg *trehalase*, *amino acid* and *glucose transporters*, *triacylglycerol transfer proteins*).

In the set of 694 orthologs identified as evolving under relaxed selection in monoecious species and intensified selection in heteroecious species, two of the five GO terms enriched in the gene set identified by Codeml analysis, were significantly over-represented: apical plasma membrane and hydrolase activity acting on glycosyl bonds, supported by 34 and 18 orthologs, respectively (Table S3). After removing redundancy between the two terms, 47 genes were retained, of which 40 had a predicted function. Together, these genes suggest roles in environmental sensing and signaling (*Tollo*, *Toll-like receptors*, *presenilin*, *Epac*, *Ncc69*, *ClC-a*, *cac*); cytoskeletal regulation and morphogenesis (*RhoGEF2*, *RhoGAP19D*, *dia*, *mew*, *stan*, *mys*, *rst*, *Shroom*, *papillote*) potentially associated with morph determination; metabolism and nutrient processing (*fatty acyl-CoA reductase*, *glycogen debranching enzyme*, *maltases*, *trehalase-like proteins*, *beta-glucuronidase*, *mannosidases*, *chitinase*, *amino acid*, and *phospholipid transporters*), and stress response and homeostasis (*Edem2*, *Fbl6*, and *Drip*), which may contribute to acclimation to different host plants.

To complement the GO term analysis, we examined the 10 most significant orthologs from both the Codeml and RELAX analyses. Of these, eight overlapped between Codeml and both types of RELAX analyses (genes either relaxed in monoecious species or intensified in heteroecious species), while nine overlapped between Codeml and at least one of the two RELAX analyses (Table S4). After accounting for shared entries, this resulted in a total of 12 orthologs, of which 10 were annotated (Table S4). The identified genes can be assigned to three main functional categories: transcriptional regulation (*Polr3F*, *CCR4-NOT subunit 1-like*), membrane dynamics and signaling (*VPS13A-like*, *endothelin-converting enzyme 2-like*, *toll-like protein*, and *piezo-type ion channel*), and cytoskeletal/motor-associated proteins (*bent*, *kl-3*, and *KIF-binding protein*). Together, these genes highlight roles in gene regulation, signaling, and cellular functions including motility and transport.

## Discussion

### Multiple Evolutionary Shifts Between Heteroecy and Monoecy in Aphids

Resolving the phylogenetic relationships among aphid lineages has long been hindered by conflicting topologies at deeper nodes, limiting our understanding of life cycle evolution in the group. Although recent studies (Hardy et al. 2022; Smith et al. 2022; Jousselin et al. 2024) have yielded increasingly consistent results concerning the placement of major subfamilies, certain relationships remain unresolved. Notably, the phylogenetic position of Calaphidinae varies across studies: some place it as sister to Aphidinae (Nováková et al. 2013; Owen and Miller 2022; Jousselin et al. 2024), while others—including our own—recover it closer to Chaitophorinae and Drepanosiphinae (Hardy et al. 2022). This discrepancy is particularly relevant because Calaphidinae, Chaitophorinae, and Drepanosiphinae are entirely monoecious, whereas Aphidinae include both monoecious and heteroecious species. Our results also support some recent findings that Eriosomatinae—a subfamily largely composed of heteroecious species—is actually paraphyletic (Nováková et al. 2013; Hardy et al. 2022; Jousselin et al. 2024). Consequently, these unresolved relationships complicate efforts to infer the evolutionary origins of heteroecy. In addition, the majority of available aphid genomes (approximately two-thirds) are from members of the Aphidinae, and then current genomic resources are still insufficient to rigorously test alternative scenarios for the evolution of host alternation across families. Nonetheless, our phylogeny confirms that heteroecy and monoecy are distributed across diverse aphid lineages among the Aphididae family, including within genera such as *Aphis*, *Brachycaudus*, and *Myzus*. Consistent with prior studies, this pattern suggests that host alternation likely evolved independently on several occasions and remains a labile trait in aphids (von Dohlen and Moran 2000; Jousselin et al. 2010; Ortiz-Rivas and Martínez-Torres 2010; Hardy et al. 2015).

### Life Cycle Simplification Leads to Relaxed Evolution

We observed higher rates of evolution in monoecious than in heteroecious species, with a significant part of the genes showing increased dN/dS in monoecious species. In several other groups, evolutionary transitions to new environments coincide with a reduction in the effective size of populations (*N_e_*), leading to less effective purifying selection, hence higher dN/dS (Charlesworth 2009). *N_e_* reduction thus explains part of the increase in dN/dS following the gain of sociality in spiders and termites (Tong et al. 2022; Roux et al. 2024), the evolution of a parasitic lifestyle in ants (Schrader et al. 2021) or endosymbiosis in bacteria (Siozios et al. 2024), the transition to more complex body architectures in algae (Uthanumallian et al. 2024) or the adaptation to aquatic lifestyle in mammals (Farina et al. 2024). In aphids, the observed increase in dN/dS associated with monoecy is unlikely to emerge from a decrease in *N_e_*. This is because heteroecious species, which must undertake costly and risky migration in spring and fall to complete their annual life cycle on alternative host plants (Ward et al. 1998), are more likely to experience recurrent population bottlenecks, leading to reduced *N_e_* compared to monoecious species. Other factors commonly used as correlates of *N_e_*, such as longevity or body weight (eg Chen et al. 2017; Farina et al. 2024), are unlikely to differentially affect the *N_e_* of monoecious and heteroecious aphids, as the two groups show no known differences in these traits (Dixon 1998). Interestingly, we revealed that most genes that experienced a shift in selection regime along the terminal branches leading to monoecy underwent relaxed selection. The opposite pattern was observed on branches leading to heteroecy, where the majority showed intensified selection (mostly via purifying selection). These divergent evolutionary outcomes are consistent with the expectations that (i) transitions to monoecy enable the relaxation of selective pressures on genes associated with host alternation and (ii) heteroecy imposes strong genetic constraints (Moran 1988, 1990; Hardy et al. 2015). The large proportion of genes exhibiting significant signs of relaxed selection (∼8% here) aligns with findings from other genomic studies on evolutionary transitions, although the mechanisms and evolutionary forces that induce them are different. As example, up to 20% of genes were subject to relaxed purifying selection following the shift from terrestrial to aquatic lifestyles in mammals (Farina et al. 2024), while 32% showed a similar pattern in termites after the evolution of sociality (Roux et al. 2024).

The relaxation of constraints on a large portion of the genome owing to the reduction of life cycle complexity may provide raw material for evolutionary innovation and the emergence of novel traits (Draghi et al. 2024). Moreover, reducing the need for tradeoffs associated with alternating between two host plants in heteroecy allows formerly pleiotropic genes to more readily reach optimal states under monoecy, where only a single host is used. In salamanders for instance, transitions from a biphasic (alternating aquatic and terrestrial life) to a uniphasic lifestyle (exclusively terrestrial or aquatic) lead to much greater rates of phenotypic evolution (Bonett and Blair 2017; Bonett et al. 2018). The higher rate of speciation observed in monoecious aphid lineages (Blackman and Eastop 1984; Moran 1992; Hardy et al. 2015) may stem from the release of raw genetic material due to relaxed selection, which enhances evolutionary flexibility, alongside the removal of pleiotropic constraints. This can facilitate host shifts, especially through adaptation onto novel herbaceous plants, fostering ecological specialization and ultimately reproductive isolation via ecological speciation (Peccoud et al. 2009, 2010). A more compelling explanation, however, is the ecological opportunity presented by herbaceous plants, that have more recently diversified and are abundant in temperate regions where most Aphidinae occur (Heie 1994; von Dohlen and Moran 2000). If speciation proceeds primarily via host plant shifts, then losing the primary host will facilitate diversification onto herbaceous plants. In contrast, aphid lineages tied to a common primary host, as in heteroecious species, are constrained by recurrent mating on the same plant, limiting genetic divergence, and preventing colonization of new herbaceous niches (Moran 1992).

### Genetic and Molecular Insights into Host Alternation in Aphids

The molecular basis of host alternation in aphids remains poorly understood, despite its ecological and evolutionary importance. So far, only two transcriptomic studies have examined gene expression changes between seasonal hosts in heteroecious species (Cui et al. 2017; Thorpe et al. 2020), suggesting potential roles for nutrient sensing, suppression of plant immune response, and detoxification of plant secondary metabolites. However, these studies offer limited insight into the broader molecular pathways underlying this complex life cycle strategy, which likely involves multiple layers of genetic, epigenetic, and environmental regulation.

Our genome-wide analyses identified key genetic signatures linked to host alternation in aphids. By pinpointing orthologs under relaxed selection in monoecious species—where host alternation has been lost—we proposed gene functions likely essential to this trait. These include roles in environmental sensing, neural function, and signalization, muscle development and organization, metabolite transport, and membrane remodeling. Their involvement suggests a coordinated system for environmental cue perception, physiological, and neural remodeling, as well as host recognition and nutritional adjustment, supporting successful shifts between distinct plant environments via specialized migrant morphs. Notably, several candidate genes (eg Toll-like receptors, transcriptional regulators, and ion transporters) were identified by both Codeml and RELAX analyses, underscoring their likely importance in the host alternation machinery. Our results thus move beyond single-species transcriptomics and offer a broader molecular framework for understanding this complex trait, paving the way for functional studies of the identified genes and the signaling pathways and regulatory networks.

## Conclusion

Our results offer three key insights into the evolution of complex life cycles. First, they provide direct evidence that reduction in life cycle complexity leaves genome-wide, predictable signatures of molecular evolution. Second, the functional categories enriched under relaxed selection suggest that host alternation is underpinned by a complex genetic architecture governing developmental plasticity, migration and host plant acclimation. The erosion of this architecture in monoecious species illustrates how ecological simplification triggers coordinated functional and genomic decay. Third, although heteroecy may confer short-term ecological advantages, it proves to be an unstable life-cycle strategy over the long term, as indicated by its low prevalence in aphids relative to monoecy. Our results provide empirical support for the view that this instability arises from genetic constraints, likely associated to developmental complexity and over-specialization.

## Materials and Methods

### Genomic Resources and Inference of Orthologs

We downloaded 46 complete aphid genomes from different depositories (see Table S1 for genome information). Of these genomes, 22 are from heteroecious species and 24 from monoecious species (Blackman and Eastop 1984). This dataset provides a good representation of the Aphidinae subfamily—the most species-rich subfamily—with 31 species. We used TOGA 1.1.7 (Kirilenko et al. 2023) to infer orthologs between the alignments of these 46 genomes. In brief, TOGA leverages a reference genome and its corresponding annotation to identify orthologs in query genomes. We selected the *Rhopalosiphum padi* genome and annotation (Assembly GCF_020882245.1) as the reference, chosen for its high-quality assembly and annotation. To generate the necessary pairwise alignment inputs for TOGA, each query genome was aligned against the *R. padi* reference using the whole-genome alignment tool progressive CACTUS v2.8.4 (Armstrong et al. 2020) with default parameters. TOGA was subsequently executed using these alignment chains, the reference genome, and the reference annotation as inputs. The analysis was run with the following parameters: –cesar_jobs_num 400, –cb 10,50,100, and –chain_jobs_num 20, while all other parameters were maintained at their default settings. For inferred single-copy orthologous genes where multiple isoform sequences were identified across the dataset, the isoform sequence identified in the most query species was retained. Finally, the protein sequences and coding DNA sequences (CDS) of the inferred orthologs were retrieved.

### Phylogenomic Tree Construction

We constructed a phylogenetic tree of the 46 genomes by using protein sequence files of single copy orthologs recovered from the TOGA results. A multiple alignment of the orthologs was produced with t_coffee v13.46.0.919e8c6b (Notredame et al. 2000) using the base parameters. These multiple-alignments were trimmed with ClipKit 2.3.0 (Steenwyk et al. 2020) using the “gappy” method to remove all positions containing at least one gap, with a gappyness value of 0.02, ie <1/46 (where 46 is the number of species). The phylogenetic tree was constructed by maximum-likelihood method with IQ-TREE 2.3.6 (Minh et al. 2020) from the concatenation of 1491 protein alignments. Optimization of the substitution models and the heterogeneity rate were carried out automatically using the ModelFinder option, resulting in the use of the model “Q.plant + F + R5” for tree construction. Bootstrap values to estimate branch support were set at 1000.

### Preparation of Alignments for Evolutionary Analysis

To test whether the patterns of gene evolution differ between monoecy and heteroecy, we restricted our analyses to the Aphidinae subfamily, which is the best represented in terms of genome number (*n* = 30). *Sitobion avenae* genome was removed from the following analyses due to insufficient divergence from *Sitobion miscanthi* one (Mathers et al. 2023). Only single-copy orthologs shared by at least 50% of the 15 monoecious species and 50% of the 15 heteroecious species of our Aphidinae genomes were used. CDS alignments were recovered from the TOGA output. The multiple alignments were trimmed with ClipKit 2.3.0 as above but using a codon-based method. The gappyness was set to <2 × (1/number of sequences) to keep only species-specific gaps. Sequences were homogenized by replacing all ambiguous nucleotides with “N” in order to avoid compatibility problems with subsequent bioinformatic analyses. A final stage of alignment cleaning was carried out in order to remove extremely divergent sequences, which could result from sequencing, alignment, or orthogroup inference errors. We have removed all sequences whose z-score of the divergence rate (number of sites differing from the consensus sequence over the total number of sites) is outside the interval [−2.5; 2.5]. This resulted in the removal of 0.33% of the sequences. The consensus sequences were obtained with the most common nucleotide for each site. Gene trees were constructed in the same way as the species tree, using IQ-TREE with ModelFinder and 1000 bootstraps.

### Estimation of Ortholog dN/dS Ratios

We used Codeml from PAML 4.10.6 (Yang 2007) to build two different branch models: a null model M_0_ with two dN/dS ratios and an alternative model M_1_ with three dN/dS ratios. The null model (M_0_) has a dN/dS ratio for all the terminal branches (those leading to the species) and a second dN/dS ratio for the rest of the branches (the background). The alternative model M_1_ has a dN/dS ratio for the background, a second ratio for the terminal branches leading to monoecious species, and a third ratio for the terminal branches leading to heteroecious species. This approach allows us to test whether a model that assumes different dN/dS ratios depending on the life cycle provides a better fit to the observed data. For this, we ran Codeml on the CDS alignments and gene trees previously produced with the following parameters: seqtype = 1, CodonFreq = 3, and model = 2. The estimated ratios and the Log of the model Likelihood (LnL) were then extracted from the results. For each ortholog, we performed a likelihood ratio test (LRT) to identify which model (M_0_ vs M_1_) better fits the data and tested its significance with a one-degree-of-freedom χ^2^ test. Finally, *P*-values were corrected for multiple testing using the BH method (Benjamini and Hochberg 1995). Using an adjusted *P*-value cutoff of 0.05, this gave us a list of orthologs that best fit the M_1_ model, ie that had different dN/dS depending on the type of life cycle.

### Characterization of the Selection Regime

We used RELAX 4.5 from HyPhy 2.5.63 (Wertheim et al. 2015) to infer if orthologs went through stronger intensified or relaxed selection in tested branches compared to a reference set. Terminal branches leading to monoecious species were designated as test branches, whereas those leading to heteroecious species served as reference branches (the reverse comparison was also performed; see below). All remaining internal branches were designated as unclassified—and thus excluded from parameter estimation—because the life-cycle status of these deeper nodes cannot be reliably inferred. The RELAX branch-site model assesses differences in the distribution of dN/dS ratios across sites between reference and test branches. Under relaxed selection, dN/dS ratios in the test branches tend to approach 1, indicating a relaxation of selective pressure. Conversely, under intensified selection, the distribution of dN/dS ratios diverges from 1, reflecting stronger purifying selection (lower values) on some sites and stronger positive selection (higher values) on others. As a result, the dN/dS distribution on test branches shifts toward both extremes. RELAX quantifies this effect using a parameter called the *k*-value: values <1 suggest relaxed selection on test branches, whereas values >1 indicate intensified selection. A LRT is then used to compare the fit of this model against a null model (M_0_) in which both test and reference branches share the same dN/dS distribution. The *P*-values are then corrected for multiple testing with BH method (Benjamini and Hochberg 1995), with the cutoff set at 0.05. We ran RELAX on the same CDS alignments and gene trees previously used for the Codeml analyses. We implemented two models: in the first (M_M_), the test branches were the terminal branches leading to monoecious species; in the second (M_H_), the test branches were those leading to heteroecious species. For each type of life cycle, orthologs were thus assigned to one of those classes: (i) orthologs that better fit the null M_0_ models (ie no evidence of change in selective regimes between the two categories of terminal branches), (ii) orthologs that better fit the alternative model and evolve under relaxed selection in the test branches, (iii) orthologs that better fit the alternative model and evolve under intensified selection in the tested branches. Finally, the results obtained with Codeml and RELAX were compared to determine whether both methods produced convergent results and to identify a list of best candidate orthologs. A candidate ortholog, in this context, refers to a gene potentially involved in host alternation as it exhibits signs of relaxed selection in monoecious species.

### Gene Function and GO Enrichment

We examined the functions and GO enrichment among orthologs identified by Codeml and RELAX analyses as evolving differently depending on the life cycle. Two gene subsets were tested for enrichment: (i) orthologs showing higher dN/dS in monoecious species than in heteroecious species, based on Codeml results, and (ii) orthologs evolving under relaxed selection in monoecious species and intensified selection in heteroecious species, as identified by RELAX. GO term annotation was performed using FANTASIA v1 pipeline (Functional ANnoTAtion based on embedding space SImilArity, Martínez-Redondo et al. 2025) with default parameters, on the predicted protein sequences from *R. padi* reference annotation. The longest isoform sequence identified for each *R. padi* gene served as the input for this analysis. For the 351 genes not annotated by FANTASIA v1, we retrieved their existing GO annotations available from NCBI (GCF_020882245.1-RS), which were originally derived from InterProScan results. GO term enrichment analysis was performed with the enrichGO function of the clusterProfiler 4.6.2 (Xu et al. 2024) package in R v4.2.2. GO terms were considered significantly enriched when *P*-value adjusted with the BH method (Benjamini and Hochberg 1995) was below 0.05. The background set for enrichment comprised 9,304 annotated orthologs.

## Supplementary Material

msaf307_Supplementary_Data

## Data Availability

The data underlying this article are available in the article and in its online material.

## References

[msaf307-B1] Armstrong J et al Progressive Cactus is a multiple-genome aligner for the thousand-genome era. Nature. 2020:587:246–251. 10.1038/s41586-020-2871-y. 33177663 PMC7673649

[msaf307-B2] Benjamini Y, Hochberg Y. Controlling the false discovery rate: a practical and powerful approach to multiple testing. J. R. Stat. Soc. Ser. B Stat. Methodol. 1995:57:289–300. 10.1111/j.2517-6161.1995.tb02031.x.

[msaf307-B3] Blackman RL, Eastop VF. Aphids on the world's Crops: an identification and information guide. J. Wiley & sons; 1984.

[msaf307-B4] Bonett RM, Blair AL. Evidence for complex life cycle constraints on salamander body form diversification. Proc Natl Acad Sci U S A. 2017:114:9936–9941. 10.1073/pnas.1703877114. 28851828 PMC5604006

[msaf307-B5] Bonett RM, Phillips JG, Ledbetter NM, Martin SD, Lehman L. Rapid phenotypic evolution following shifts in life cycle complexity. Proc. R. Soc. B Biol. Sci. 2018:285:20172304. 10.1098/rspb.2017.2304. PMC5805936 29343600

[msaf307-B6] Charlesworth B . Effective population size and patterns of molecular evolution and variation. Nat Rev Genet. 2009:10:195–205. 10.1038/nrg2526. 19204717

[msaf307-B7] Chen J, Glémin S, Lascoux M. Genetic diversity and the efficacy of purifying selection across plant and animal species. Mol Biol Evol. 2017:34:1417–1428. 10.1093/molbev/msx088. 28333215

[msaf307-B8] Choi H, Shin S, Jung S, Clarke DJ, Lee S. Molecular phylogeny of macrosiphini (Hemiptera: Aphididae): an evolutionary hypothesis for the pterocomma-group habitat adaptation. Mol Phylogenet Evol. 2018:121:12–22. 10.1016/j.ympev.2017.12.021. 29253532

[msaf307-B9] Cui N, Yang P-C, Guo K, Kang L, Cui F. Large-scale gene expression reveals different adaptations of *Hyalopterus persikonus* to winter and summer host plants. Insect Sci. 2017:24:431–442. 10.1111/1744-7917.12336. 28547891

[msaf307-B10] Dixon AFG . Aphid ecology an optimization approach. Springer Science & Business Media; 1998.

[msaf307-B11] Draghi J, Ogbunugafor CB, Zaman L, Parsons TL. 2024 Jul 9. Relaxed selection can speed the evolution of complex adaptations [preprint]. bioRxiv. 2024.07.09.602773. 10.1101/2024.07.09.602773.

[msaf307-B12] Eastment RV, Wong BBM, McGee MD. Convergent genomic signatures associated with vertebrate viviparity. BMC Biol. 2024:22:34. 10.1186/s12915-024-01837-w. 38331819 PMC10854053

[msaf307-B13] Emden HF, Harrington R. Aphids as crop pests. 1st ed. CABI; 2007.

[msaf307-B14] Farina BM, Latrille T, Salamin N, Silvestro D, Faurby S. 2024 Sep 21. Widespread selection relaxation in aquatic mammals [preprint]. bioRxiv. 2024.09.18.613479. 10.1101/2024.09.18.613479.

[msaf307-B15] Hardy NB et al Geographic isolation drives speciation in Nearctic aphids. Commun Biol. 2022:5:796. 10.1038/s42003-022-03771-5. 35941371 PMC9360434

[msaf307-B16] Hardy NB, Peterson DA, von Dohlen CD. The evolution of life cycle complexity in aphids: ecological optimization or historical constraint? Evolution. 2015:69:1423–1432. 10.1111/evo.12643. 25787153

[msaf307-B17] Heie O . Why are there so few aphid species in the temperate areas of the southern hemisphere? Eur J Entomol. 1994:91:127–133.

[msaf307-B18] Jousselin E et al Discordance between mitochondrial, nuclear, and symbiont genomes in aphid phylogenetics: who is telling the truth? Zool J Linn Soc. 2024:201:zlae098. 10.1093/zoolinnean/zlae098.

[msaf307-B19] Jousselin E, Genson G, Coeur d’acier A. Evolutionary lability of a complex life cycle in the aphid genus *Brachycaudus*. BMC Evol Biol. 2010:10:295. 10.1186/1471-2148-10-295. 20920188 PMC2958166

[msaf307-B20] Kirilenko BM et al Integrating gene annotation with orthology inference at scale. Science. 2023:380:eabn3107. 10.1126/science.abn3107. 37104600 PMC10193443

[msaf307-B21] Leulier F, Lemaitre B. Toll-like receptors—taking an evolutionary approach. Nat Rev Genet. 2008:9:165–178. 10.1038/nrg2303. 18227810

[msaf307-B22] Liedtke HC, Wiens JJ, Gomez-Mestre I. The evolution of reproductive modes and life cycles in amphibians. Nat Commun. 2022:13:7039. 10.1038/s41467-022-34474-4. 36396632 PMC9672123

[msaf307-B23] Mackenzie A, Dixon AFG. Host alternation in aphids: constraint versus optimization. Am Nat. 1990:136:132–134. 10.1086/285086.

[msaf307-B24] Mackenzie A, Dixon AFG. An ecological perspective of host alternation in aphids (Homoptera: Aphidinea: Aphididae). Entomol Gen. 1991:16:265–284. 10.1127/entom.gen/16/1991/265.

[msaf307-B25] Martínez-Redondo GI et al FANTASIA leverages language models to decode the functional dark proteome across the animal tree of life. Commun Biol. 2025:8:1227. 10.1038/s42003-025-08651-2. 40813894 PMC12354702

[msaf307-B26] Mathers TC et al Hybridisation has shaped a recent radiation of grass-feeding aphids. BMC Biol. 2023:21:157. 10.1186/s12915-023-01649-4. 37443008 PMC10347838

[msaf307-B27] McEdward LR . Adaptive evolution of larvae and life cycles. Semin Cell Dev Biol. 2000:11:403–409. 10.1006/scdb.2000.0193. 11145868

[msaf307-B28] Minh BQ et al IQ-TREE 2: new models and efficient methods for phylogenetic inference in the genomic era. Mol Biol Evol. 2020:37:1530–1534. 10.1093/molbev/msaa015. 32011700 PMC7182206

[msaf307-B29] Moran NA . The evolution of host-plant alternation in aphids: evidence for specialization as a dead end. Am Nat. 1988:132:681–706. 10.1086/284882.

[msaf307-B30] Moran NA . Aphid life cycles: two evolutionary steps. Am Nat. 1990:136:135–138. 10.1086/285087.

[msaf307-B31] Moran NA . The evolution of aphid life cycles. Annu Rev Entomol. 1992:37:321–348. 10.1146/annurev.en.37.010192.001541.

[msaf307-B32] Moran NA . Adaptation and constraint in the complex life cycles of animals. Annu Rev Ecol Syst. 1994:25:573–600. 10.1146/annurev.es.25.110194.003041.

[msaf307-B33] Notredame C, Higgins DG, Heringa J. T-coffee: a novel method for fast and accurate multiple sequence alignment1. J Mol Biol. 2000:302:205–217. 10.1006/jmbi.2000.4042. 10964570

[msaf307-B34] Nováková E et al Reconstructing the phylogeny of aphids (Hemiptera: aphididae) using DNA of the obligate symbiont Buchnera aphidicola. Mol Phylogenet Evol. 2013:68:42–54. 10.1016/j.ympev.2013.03.016. 23542003

[msaf307-B35] Ortiz-Rivas B, Martínez-Torres D. Combination of molecular data support the existence of three main lineages in the phylogeny of aphids (Hemiptera: Aphididae) and the basal position of the subfamily Lachninae. Mol Phylogenet Evol. 2010:55:305–317. 10.1016/j.ympev.2009.12.005. 20004730

[msaf307-B36] Owen CL, Miller GL. Phylogenomics of the Aphididae: deep relationships between subfamilies clouded by gene tree discordance, introgression and the gene tree anomaly zone. Syst Entomol. 2022:47:470–486. 10.1111/syen.12542.

[msaf307-B37] Peccoud J et al Evolutionary history of aphid-plant associations and their role in aphid diversification. C R Biol. 2010:333:474–487. 10.1016/j.crvi.2010.03.004. 20541159

[msaf307-B38] Peccoud J, Ollivier A, Plantegenest M, Simon J-C. A continuum of genetic divergence from sympatric host races to species in the pea aphid complex. Proc Natl Acad Sci U S A. 2009:106:7495–7500. 10.1073/pnas.0811117106. 19380742 PMC2678636

[msaf307-B39] Peoples N, Burns MD, Mihalitsis M, Wainwright PC. Evolutionary lability of a key innovation spurs rapid diversification. Nature. 2025:639:962–967. 10.1038/s41586-025-08612-z. 40011783

[msaf307-B40] Ponton F et al The complex interactions between nutrition, immunity and infection in insects. J Exp Biol. 2023:226:jeb245714. 10.1242/jeb.245714. 38095228

[msaf307-B41] Roux C, Ha A, Weyna A, Lode M, Romiguier J. The impact of social complexity on the efficacy of natural selection in termites. Peer Community J. 2024:4:e101. 10.24072/pcjournal.476.

[msaf307-B42] Schrader L et al Relaxed selection underlies genome erosion in socially parasitic ant species. Nat Commun. 2021:12:2918. 10.1038/s41467-021-23178-w. 34006882 PMC8131649

[msaf307-B43] Shih P-Y, Sugio A, Simon J-C. Molecular mechanisms underlying host plant specificity in aphids. Annu Rev Entomol. 2023:68:431–450. 10.1146/annurev-ento-120220-020526. 36228134

[msaf307-B44] Siozios S et al Genome dynamics across the evolutionary transition to endosymbiosis. Curr Biol. 2024:34:5659–5670.e7. 10.1016/j.cub.2024.10.044. 39549700

[msaf307-B45] Smith TE, Li Y, Perreau J, Moran NA. Elucidation of host and symbiont contributions to peptidoglycan metabolism based on comparative genomics of eight aphid subfamilies and their *Buchnera*. PLOS Genet. 2022:18:e1010195. 10.1371/journal.pgen.1010195. 35522718 PMC9116674

[msaf307-B46] Steenwyk JL, Iii TJB, Li Y, Shen X-X, Rokas A. ClipKIT: a multiple sequence alignment trimming software for accurate phylogenomic inference. PLOS Biol. 2020:18:e3001007. 10.1371/journal.pbio.3001007. 33264284 PMC7735675

[msaf307-B47] Thorpe P, Escudero-Martinez CM, Eves-van den Akker S, Bos JIB. Transcriptional changes in the aphid species *Myzus cerasi* under different host and environmental conditions. Insect Mol Biol. 2020:29:271–282. 10.1111/imb.12631. 31846128 PMC7317760

[msaf307-B48] Tong C, Avilés L, Rayor LS, Mikheyev AS, Linksvayer TA. Genomic signatures of recent convergent transitions to social life in spiders. Nat Commun. 2022:13:6967. 10.1038/s41467-022-34446-8. 36414623 PMC9681848

[msaf307-B49] Uthanumallian K et al Genome-wide patterns of selection-drift variation strongly associate with organismal traits across the green plant lineage. Genome Res. 2024:34:1130–1139. 10.1101/gr.279002.124. 39209552 PMC11444171

[msaf307-B50] von Dohlen CD, Moran NA. Molecular data support a rapid radiation of aphids in the Cretaceous and multiple origins of host alternation. Biol. J. Linn. Soc. 2000:71:689–717. 10.1006/bijl.2000.0470.

[msaf307-B51] von Dohlen CD, Rowe CA, Heie OE. A test of morphological hypotheses for tribal and subtribal relationships of Aphidinae (Insecta: Hemiptera: Aphididae) using DNA sequences. Mol Phylogenet Evol. 2006:38:316–329. 10.1016/j.ympev.2005.04.035. 16368250

[msaf307-B52] Ward SA, Leather SR, Pickup J, Harrington R. Mortality during dispersal and the cost of host-specificity in parasites: how many aphids find hosts? J Anim Ecol. 1998:67:763–773. 10.1046/j.1365-2656.1998.00238.x.

[msaf307-B53] Wertheim JO, Murrell B, Smith MD, Kosakovsky Pond SL, Scheffler K. RELAX: detecting relaxed selection in a phylogenetic framework. Mol Biol Evol. 2015:32:820–832. 10.1093/molbev/msu400. 25540451 PMC4327161

[msaf307-B54] Xu S et al Using clusterProfiler to characterize multiomics data. Nat Protoc. 2024:19:3292–3320. 10.1038/s41596-024-01020-z. 39019974

[msaf307-B55] Yang Z . PAML 4: phylogenetic analysis by maximum likelihood. Mol Biol Evol. 2007:24:1586–1591. 10.1093/molbev/msm088. 17483113

[msaf307-B56] Zhao X et al Genomic signatures associated with the evolutionary loss of egg yolk in parasitoid wasps. Proc Natl Acad Sci U S A. 2025:122:e2422292122. 10.1073/pnas.2422292122. 40232796 PMC12036997

