# Supplementary material for: Life Cycle Simplifications in Aphids Drive Changes in Evolutionary Rates and Selection Regimes
Source: Mol Biol Evol. 2025 Dec 1;42(12):msaf307. doi: 10.1093/molbev/msaf307 (PMC12696414; doi:10.1093/molbev/msaf307)
Supplement: msaf307_Supplementary_Data [file msaf307_supplementary_data.zip › Suplementary_Figures.docx]

**Supplementary data**

**Table S1**: Features, accessions and taxonomic information of the aphid genomic resources.

**Table S2**: Summary of Codeml and RELAX analyses for each of the 9304 orthologs. This table also includes putative function annotation of each gene.

**Table S3**: GO enrichment obtained with ClusterProfiler R package for two candidate ortholog sets, compared to a background set of 9304 orthologs. These sets included the orthologs with higher dN/dS in monoecious than in heteroecious species from the Codeml analyses (first sheet) and the orthologs identified from the RELAX analyses as evolving under relaxed selection in monoecious species and intensified selection in heteroecious species (third sheet). The redundancy of the genes contributing to the enriched GO terms and their functional annotation is also joined for the Codeml set (second sheet) and RELAX set (fourth sheet).

**Table S4**: Annotation of the 10 most significant orthologs with higher dN/dS in monoecious species (Codeml analysis) or evolving under relaxed selection in monoecious or heteroecious species (RELAX analysis).


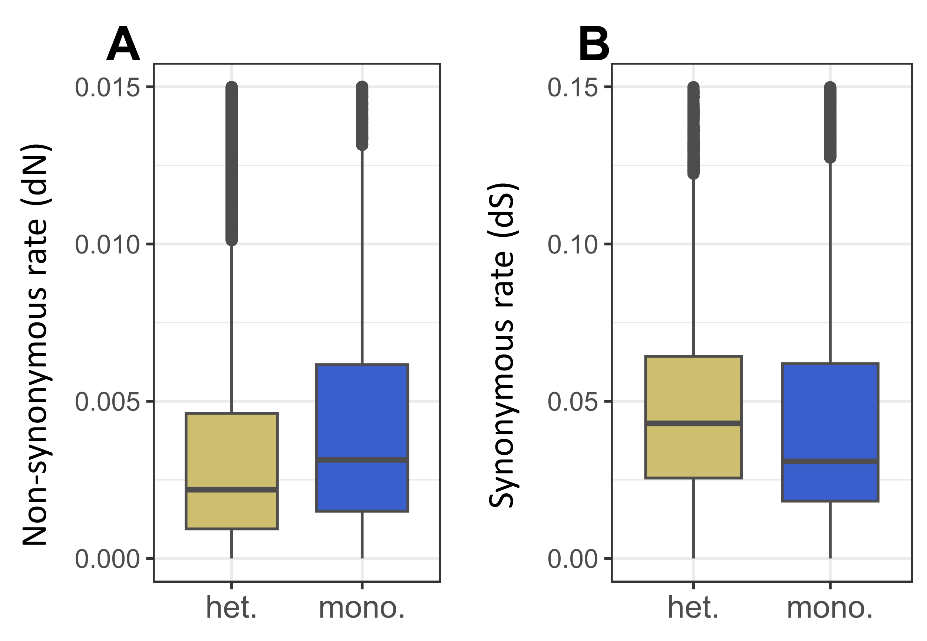


**Figure S1**. Distribution of the estimated non-synonymous A) and synonymous B) rates from terminal branches sorted by life cycle, for the 758 orthologs fitting better the Codeml M_1_ model.


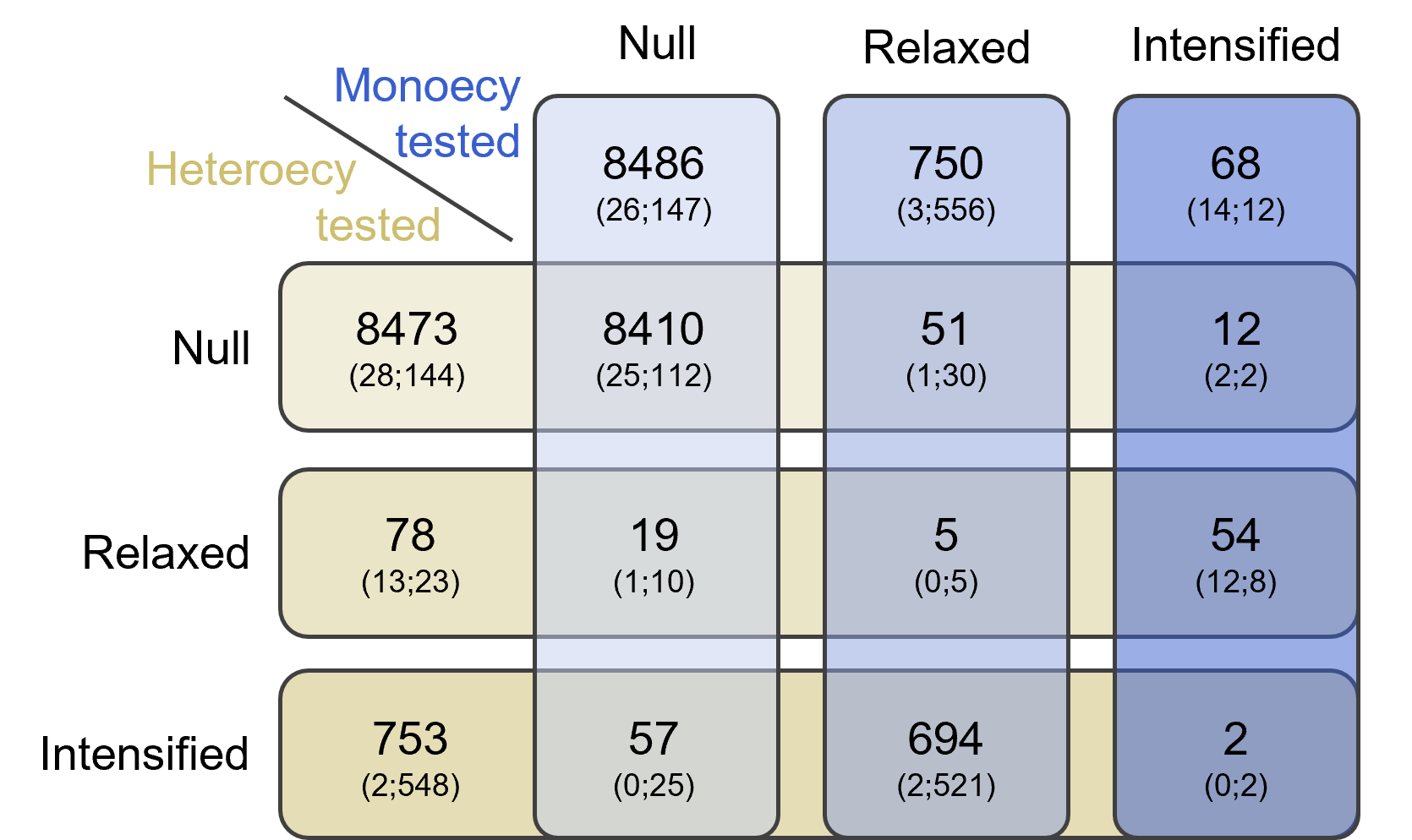


**Figure S2**. Combination of results from the RELAX and the Codeml analyses. In rows (yellow), number of genes that best fit the null model, or that show signs of relaxed or intensified selection in heteroecious terminal branches compared to terminal monoecious branches (model M_H_) following RELAX. In columns (blue), same type of results but when the RELAX test is carried out on the monoecious terminal branches compared to terminal heteroecious branches (model M_M_). The intersection of RELAX results is shown in the center of the figure. In brackets are the numbers of these genes fitting better the Codeml M_1_ model and showing higher dN/dS in heteroecious species (first value) or in monoecious species (second value).

**References of Genomics resource (Table S1)**

Biello R, Singh A, Godfrey CJ, Fernández FF, Mugford ST, Powell G, Hogenhout SA, Mathers TC. 2021. A chromosome‐level genome assembly of the woolly apple aphid, *Eriosoma lanigerum* Hausmann (Hemiptera: Aphididae). *Mol. Ecol. Resour.* 21:316–326.

Burger NFV, Nicolis VF, Botha A-M. 2025. Evaluating long-read assemblers to assemble several aphididae genomes. *Brief. Bioinform.* 26:bbaf105.

Crowley LM. 2024. The genome sequence of the sycamore periphyllus aphid, *Periphyllus acericola* (Walker, 1848). *Wellcome Open Res.* 9:676.

Crowley LM, James R. 2023. The genome sequence of the common sycamore aphid, *Drepanosiphum platanoidis* (Schrank, 1801). *Wellcome Open Res.* 8:481.

Crowley LM, McCulloch J, James R. 2024. The genome sequence of the giant willow aphid, *Tuberolachnus salignus* (Gmelin, 1790). *Wellcome Open Res.* 9:59.

Dial DT, Weglarz KM, Brunet BMT, Havill NP, von Dohlen CD, Burke GR. 2023. Whole-genome sequence of the Cooley spruce gall adelgid, *Adelges cooleyi* (Hemiptera: Sternorrhyncha: Adelgidae).McIntyre L, editor. *G3 Genes Genomes Genet.* 14:jkad224.

Feng H, Chen W, Hussain S, Shakir S, Tzin V, Adegbayi F, Ugine T, Fei Z, Jander G. 2023. Horizontally transferred genes as RNA interference targets for aphid and whitefly control. *Plant Biotechnol. J.* 21:754–768.

Garrett D, Bell JR, Morales-Hojas R, Teakle GR, Collier R, Garfield E. 2022. Utilising ecology and molecular techniques to identify alternative hosts, Green Bridges, and population structure of a serious aphid pest. *University of Wacrwick.*

Huang T, Liu Y, He K, Francis F, Wang B, Wang G. 2023. Chromosome-level genome assembly of the spotted alfalfa aphid *Therioaphis trifolii*. *Sci. Data* 10:274.

Julca I, Marcet-Houben M, Cruz F, Vargas-Chavez C, Johnston JS, Gómez-Garrido J, Frias L, Corvelo A, Loska D, Cámara F, et al. 2020. Phylogenomics identifies an ancestral burst of gene duplications predating the diversification of aphidomorpha. Battistuzzi FU, editor. *Mol. Biol. Evol.* 37:730–756.

Korgaonkar A, Han C, Lemire AL, Siwanowicz I, Bennouna D, Kopec RE, Andolfatto P, Shigenobu S, Stern DL. 2021. A novel family of secreted insect proteins linked to plant gall development. *Curr. Biol.* 31:1836-1849.e12.

Li Z, Xue AZ, Maeda GP, Li Y, Nabity PD, Moran NA. 2023. Phylloxera and aphids show distinct features of genome evolution despite similar reproductive modes. Larracuente A, editor. *Mol. Biol. Evol.* 40:msad271.

Mathers TC. 2020. Improved genome assembly and annotation of the soybean aphid (*Aphis glycines* Matsumura). *G3 GenesGenomesGenetics* 10:899–906.

Mathers TC, Mugford ST, Hogenhout SA, Tripathi L. 2020. Genome sequence of the banana aphid, *Pentalonia nigronervosa* Coquerel (Hemiptera: Aphididae) and Its Symbionts. *G3 GenesGenomesGenetics* 10:4315–4321.

Mathers TC, Wouters RHM, Mugford ST, Swarbreck D, van Oosterhout C, Hogenhout SA. 2021. Chromosome-scale genome assemblies of aphids reveal extensively rearranged autosomes and long-term conservation of the X chromosome. Ouangraoua A, editor. *Mol. Biol. Evol.* 38:856–875.

Olvera-Vazquez SG, Chen X, Mesnil A, Meslin C, Almeida-Silva F, Confais J, Bourgeois Y, Lombardi G, Lougmani C, Alix K, et al. 2025. Comprehensive annotation of olfactory and gustatory receptor genes and transposable elements revealed their evolutionary dynamics in aphids.True J, editor. *Mol. Biol. Evol.*:msaf238.

Smith TE, Li Y, Perreau J, Moran NA. 2022. Elucidation of host and symbiont contributions to peptidoglycan metabolism based on comparative genomics of eight aphid subfamilies and their Buchnera. Hughes D, editor. *PLOS Genet.* 18:e1010195.

Thorpe P, Escudero-Martinez CM, Cock PJA, Eves-van den Akker S, Bos JIB. 2018. Shared transcriptional control and disparate gain and loss of aphid parasitism genes. Moran N, editor. *Genome Biol. Evol.* 10:2716–2733.

Voronova NV, Levykina S, Warner D, Shulinski R, Bandarenka Y, Zhorov D. 2020. Characteristic and variability of five complete aphid mitochondrial genomes: *Aphis fabae mordvilkoi, Aphis craccivora, Myzus persicae, Therioaphis tenera* and *Appendiseta robiniae* (Hemiptera; Sternorrhyncha; Aphididae). *Int. J. Biol. Macromol.* 149:187–206.

Wei H-Y, Ye Y-X, Huang H-J, Chen M-S, Yang Z-X, Chen X-M, Zhang C-X. 2021. Chromosome-level genome assembly of the horned-gall aphid, *Schlechtendalia chinensis* (Hemiptera: Aphididae: Erisomatinae). *bioRxiv.* 2021.02.17.431348

Whitehead M, Karley A, Darby A. 2023. Genomic insights into clonal diversity in UK populations of the Potato aphid, *Macrosiphum euphorbiae*. *bioRxiv.* 2023.05.28.542558

Ye S, Zeng C, Liu J-F, Wu C, Song Y-F, Qin Y-G, Yang M-F. 2022. A chromosome-level genome assembly of *Neotoxoptera formosana* (Takahashi, 1921) (Hemiptera: Aphididae).Sethuraman A, editor. *G3 GenesGenomesGenetics* 12:jkac164.

Zhang S, Gao X, Wang L, Jiang W, Su H, Jing T, Cui J, Zhang L, Yang Y. 2022. Chromosome‐level genome assemblies of two cotton‐melon aphid *Aphis gossypii* biotypes unveil mechanisms of host adaption. *Mol. Ecol. Resour.* 22:1120–1134.

Zhao J, Xie L, Zhao X, Li L, Cui J, Chen J. 2024. Genome sequence of the sugarcane aphid, *Melanaphis sacchari* (Hemiptera: Aphididae).Sachs M, editor. *G3 Genes Genomes Genet.* 14:jkae223.

Zhu B, Wei R, Hua W, Li L, Zhang W, Liang P, Gao X. 2022. A High-Quality Chromosome-Level Assembly Genome Provides Insights into Wing Dimorphism and Xenobiotic Detoxification in Metopolophium Dirhodum (Walker). *Research Square.*
